# Aerobic Exercise Ameliorates Cancer Cachexia-Induced Muscle Wasting through Adiponectin Signaling

**DOI:** 10.3390/ijms22063110

**Published:** 2021-03-18

**Authors:** Makoto Morinaga, Naoki Sako, Mari Isobe, Sachiko Lee-Hotta, Hideshi Sugiura, Satoshi Kametaka

**Affiliations:** 1 Division of Biofunctional Sciences, Department of Integrated Health Sciences, Graduate School of Medicine, Nagoya University, 1-1-20 Daiko-Minami, Higashi-ku, Nagoya, Aichi 461-0047, Japan; m-morinaga-bj@morinaga.co.jp (M.M.); sako.naoki@g.mbox.nagoya-u.ac.jp (N.S.); hsugiura@met.nagoya-u.ac.jp (H.S.); 2 Division of Morphological Sciences, Kagoshima University Graduate School of Medicine and Dental Sciences, 8-35-1 Sakuragaoka, Kagoshima 890-8544, Japan; isobe.mari@kufm.kagoshima-u.ac.jp; 3 Division of Creative Physical Therapy, Field of Prevention and Rehabilitation Sciences, Department of Integrated Health Sciences, Graduate School of Medicine, Nagoya University, 1-1-20 Daiko-Minami, Higashi-ku, Nagoya, Aichi 461-0047, Japan; lee@met.nagoya-u.ac.jp

**Keywords:** cancer cachexia, muscle atrophy, aerobic exercise, adiponectin

## Abstract

Cachexia is a multifactorial syndrome characterized by muscle loss that cannot be reversed by conventional nutritional support. To uncover the molecular basis underlying the onset of cancer cachectic muscle wasting and establish an effective intervention against muscle loss, we used a cancer cachectic mouse model and examined the effects of aerobic exercise. Aerobic exercise successfully suppressed muscle atrophy and activated adiponectin signaling. Next, a cellular model for cancer cachectic muscle atrophy using C2C12 myotubes was prepared by treating myotubes with a conditioned medium from a culture of colon-26 cancer cells. Treatment of the atrophic myotubes with recombinant adiponectin was protective against the thinning of cells through the increased production of p-mTOR and suppression of LC3-II. Altogether, these findings suggest that the activation of adiponectin signaling could be part of the molecular mechanisms by which aerobic exercise ameliorates cancer cachexia-induced muscle wasting.

## 1. Introduction

Cancer cachexia is a multifactorial metabolic syndrome characterized by systemic inflammation and wasting of muscle and adipose tissue, leading to weight loss despite adequate nutritional support. Approximately 60–80% of patients with cancer show cachectic symptoms, which account for 20% of mortality [1]. Recently, it was demonstrated that inflammatory cytokines, including interleukin 6 (IL-6) and tumor necrosis factor α (TNF-α) secreted by tumor cells and peripheral tissues, induce the development of cachexia [1,2]. Such inflammatory cytokines are known to act on many organs [2,3] and cause various clinical symptoms, including anorexia, metabolic disorders, loss of adipose tissue, and skeletal muscle atrophy [1,2,3,4,5]. Cancer cachexia-induced muscle atrophy has also been reported to cause decreased physical activity, resulting in progressive muscle atrophy due to disuse and decreased quality of life (QOL) of the patients. Although the processes leading to cancer cachexia are well known in clinical settings, the precise mechanisms underlying skeletal muscle wasting, the main symptom of cancer cachexia, remain to be elucidated.

Skeletal muscle mass is maintained by the balance between synthesis and degradation of muscle proteins, and an imbalance between the synthesis and degradation of proteins is a major cause of cancer cachexia-induced muscle atrophy [1,3]. Inflammatory cytokines activate the ubiquitin-proteasome pathway, a major mechanism underlying the degradation of muscle proteins [6,7,8]. Several studies described the increased expression of the muscle RING-finger protein-1 (MuRF1) and Atrogin-1 (known as F-box protein 32, FBXO32) E3 ubiquitin ligases in atrophic muscles during cancer cachexia [8]. Furthermore, some previous studies revealed that autophagic proteolysis is involved in cancer cachexia-induced muscle atrophy [9,10,11]. In contrast, the expression of molecules related to protein synthesis, including insulin-like growth factor 1 (IGF-1) and phosphorylated mechanistic target of rapamycin (mTOR), is suppressed in cachectic muscles [12]. It was also demonstrated that redox homeostasis balances protein synthesis and degradation in skeletal muscles during cancer cachexia [13,14]. A correlation between loss of muscle mass and an increase in reactive oxygen species (ROS) was found in cancer cachectic animals [15,16]. ROS regulate autophagy by controlling various signaling pathways, such as PI3k/Akt/mTOR and p38/MAPK/p53 [14]. Furthermore, antioxidant treatment was shown to suppress ROS generation and attenuate cachexia-induced muscle atrophy [17].

Recent studies reported that aerobic exercise suppresses muscle atrophy in tumor-bearing mouse models [18,19,20,21]. In these studies, aerobic exercise caused activation of AKT/PKB and subsequent phosphorylation of mTOR, leading to activation of de novo protein synthesis, which is associated with phosphorylation of p70S6 kinase and 4EBP-1 [14]. Activation of AKT/mTOR pathway also caused decreased expression of E3 ubiquitin ligases and autophagic flux, major mechanisms for protein degradation [18,19,20]. Moreover, exercise can exert anti-oxidative effects by decreasing the intracellular ROS production and protect muscles from wasting during the cachectic condition [16]. The detailed molecular mechanisms for the diverse physiological effects of aerobic exercise, however, are still unclear.

Pharmacological treatments that mimic the effects of exercise were previously studied to elucidate the precise molecular basis of the protective effect of exercise on cachectic muscle atrophy and uncover new clinical interventions. One analog of adenosine monophosphate (AMP), in particular, 5-aminoimidazole-4-carboxamide-1-beta-D-ribofuranoside (AICAR), was shown to activate an AMP-activated protein kinase (AMPK), which successfully rescued muscle homeostasis in C26-bearing mice [20].

Adiponectin, an adipose-derived hormone, is abundant in human serum [22]. Adiponectin is known to be involved in cellular metabolism, including lipid metabolism, vascular protection, and reducing insulin resistance [22,23]. Aerobic exercise increases the serum levels of adiponectin and other adiponectin-related molecules in skeletal muscle tissues in obese and type 2 diabetic mice [22,23,24]. Adiponectin was also reported to promote the uptake of glucose and increase insulin sensitivity in skeletal muscles [25,26,27]. To date, Adiponectin receptor 1 (AdipoR1) and 2 (AdipoR2) have been identified as adiponectin-specific receptors [28]. Furthermore, T-cadherin, a cell adhesion molecule highly expressed in endothelial cells, is known to mediate adiponectin signaling [29]. Adiponectin binds to these receptors, and the adiponectin signal is transmitted through the recruitment of APPL1 (a adaptor protein, phosphotyrosine, interacting with PH domain and leucine zipper 1), which contains a pleckstrin homology (PH) domain, phosphotyrosine binding (PTB) domain, and a leucine zipper motif. The recruitment of APPL1 can increase insulin sensitivity and glucose uptake, mediated by the translocation of glucose transporter type 4 (GLUT4) to the plasma membrane [30].

Moreover, adiponectin might play a significant role in regulating skeletal muscle mass. In previous studies, adiponectin was reported to stimulate the differentiation, regeneration, or suppression of proteolysis in muscle tissues [31,32,33,34]. However, it is still unknown whether adiponectin has a protective action against cancer cachexia-induced muscle atrophy. Adiponectin was also identified as a myokine secreted by muscle cells, acting in an autocrine/paracrine manner by stimulating adiponectin receptors expressed in the muscle tissue [35]. However, no studies investigated the expression levels of adiponectin and its related molecules in cachectic muscle atrophy.

In this study, we examined whether adiponectin signaling induced by forced aerobic exercise was protective against muscle atrophy induced by cancer cachexia.

## 2. Results

### 2.1. Aerobic Exercise Suppresses Cancer Cachexia-Induced Muscle Atrophy

**Figure 1:**
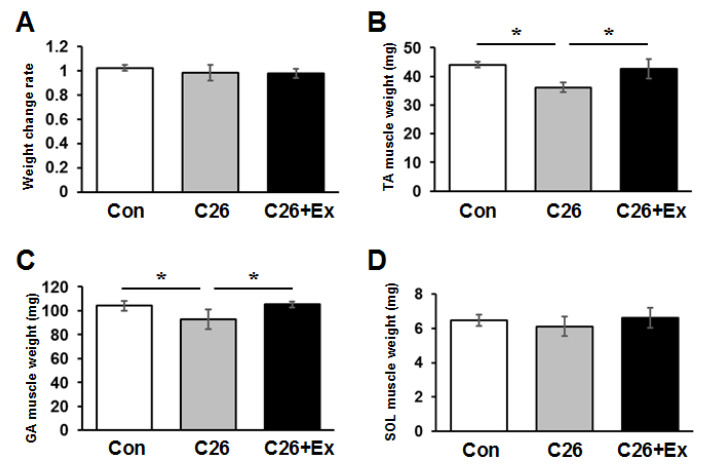
Aerobic exercise suppresses muscle atrophy. Forced running was applied to the cancer-bearing mice for 4 weeks. The ratio of tumor weight subtracted body weight at 4 weeks to the body weight at day 0 is indicated as bar graphs (**A**). Weights of (**B**) TA, (**C**) GA, and (**D**) SOL muscles from control (Con, blank bars), C26 injected (C26, gray bars) and C26 injected and aerobic exercise applied (C26+Ex, dark bars) mice are indicated. The average weight and standard deviation (SD) are indicated. Statistical analysis was carried out with the Kruskal-Wallis test followed by the Mann-Whitney U test. Asterisks: *p* < 0.05.

To examine the effect of protective intervention, especially to muscle atrophy due to cancer cachexia, we used a tumor-bearing mouse model produced by injecting C26 colon cancer cells subcutaneously [20,21]. During cancer cachexia, muscle weight decreased by approximately 16% in tibialis anterior (TA) and 12% in gastrocnemius (GA) muscles, but no significant loss of total body weight was observed (Figure 1A). Aerobic exercise via treadmill running suppressed cancer cachexia-induced muscle atrophy in both TA and GA muscles (Figure 1B,C). In contrast, aerobic exercise did not prevent weight reduction in soleus (SOL) muscle, which is known to be dominated by slow-twitch fibers (Figure 1D).

**Figure 2:**
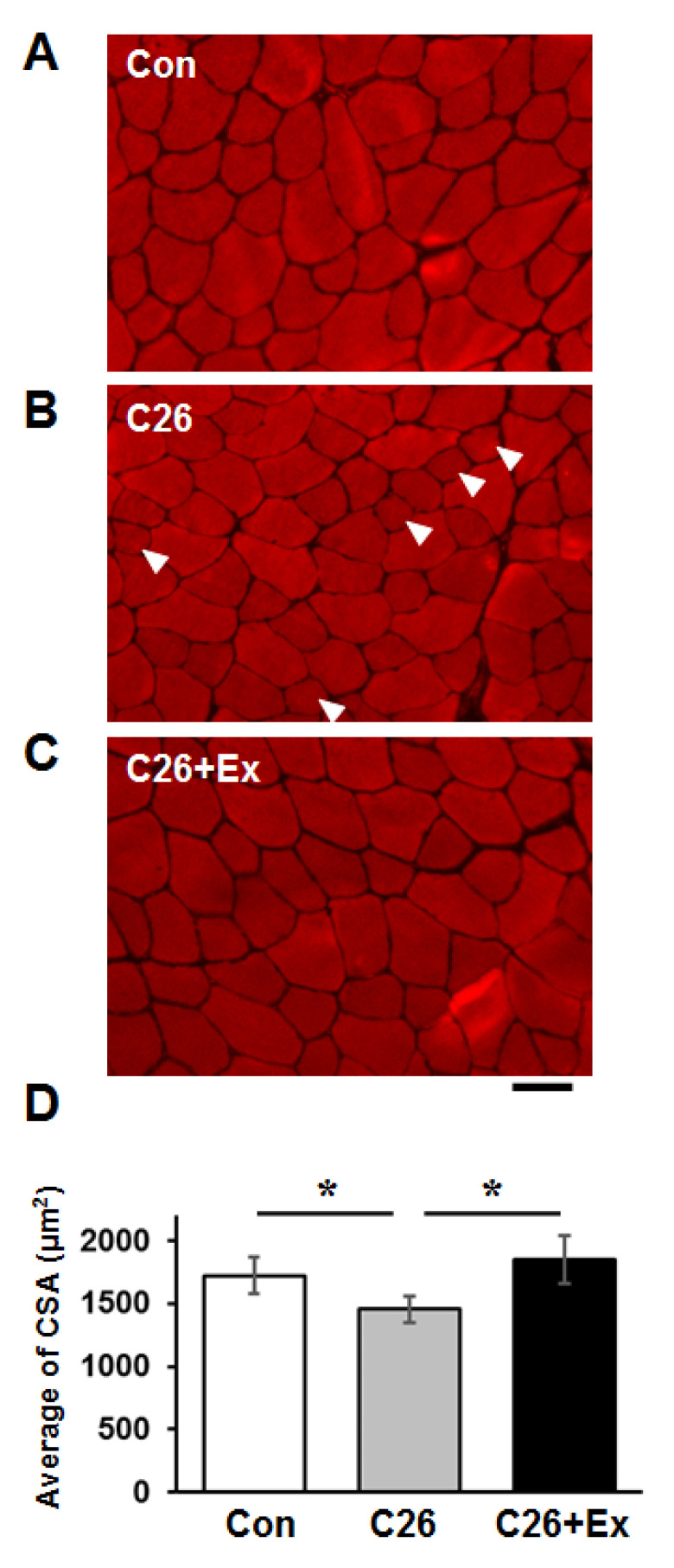
Aerobic exercise suppresses the atrophy of the skeletal C26-bearing mice. Cross-sections of TA muscles from (**A**) control (Con), (**B**) C26-bearing for 4 weeks (C26), and (**C**) C26-bearing and treadmill running applied for 4 weeks (C26+Ex) mice were stained with Alexa555-Phalloidin. Arrowheads indicate atrophic myofibers in B. (**D**) The average cross-section area of A-C is indicated. Scale bar: 50 µm. Statistical analysis was carried out with ANOVA followed by Tukey HSD tests. *: *p* < 0.05.

To further analyze the atrophic status of skeletal muscles, we conducted a histological analysis of the cross-sectional area (CSA) of the TA muscle. Accordingly, we histologically observed the appearance of the small atrophic muscle fibers in the C26 cachectic group (Figure 2B, arrowheads), and the average CSA decreased in the C26 group (Figure 2D). Aerobic exercise was shown to reduce the population of small muscle fibers and mitigate the decrease of CSA in the C26 group (Figure 2C,D). Histogram analysis of each CSA distribution showed that the population of small fibers following the injection of C26 cells was recovered by aerobic exercise. Notably, the number of fibers greater than 2400 µm^2^ in size increased to the level of control mice in the C26 group (Figure S1, Supplementary Materials). As reported previously, aerobic exercise decreased tumor size to a greater extent in the exercised mice compared to that in the non-exercised mice treated with C26 [36] (Figure S2).

### 2.2. Aerobic Exercise Enhances Protein Synthesis Signaling in Cancer Cachectic Skeletal Muscle

**Figure 3:**
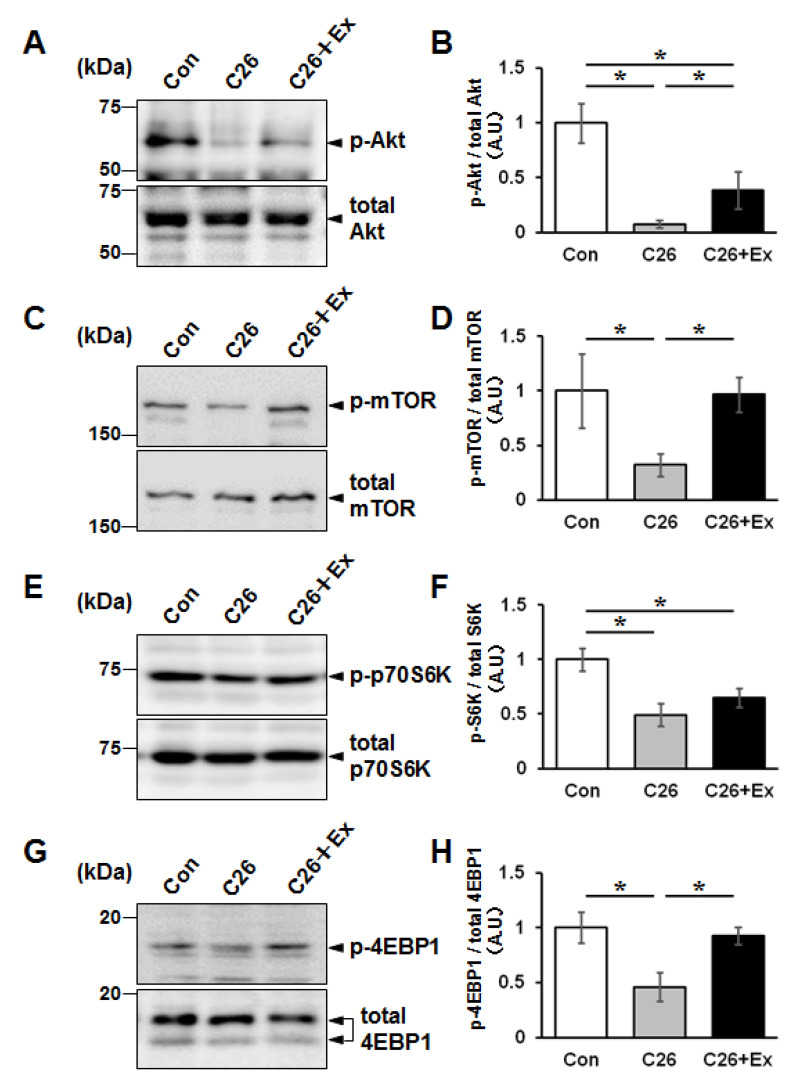
Aerobic exercise enhances the stimulation of Akt signaling cascade. Western blot analysis was performed on the TA of the mice in Figure 2 using anti-phospho-Akt (p-Akt) and pan-Akt (total Akt) antibodies (**A**), anti-phospho- and pan-mTOR (**C**), anti-phospho- and pan-p70S6 kinase (**E**), and anti-phospho and pan-4EBP-1 antibodies (**G**). The data represents the results of three independent experiments. Densitometric analyses of Western blot shown in (**A**,**C**,**E**,**G**) are presented in (**B**,**D**,**F**,**H**), respectively. The bar graphs indicate the mean relative expression ± SD. Statistical analysis was performed by Kruskal-Wallis analysis followed by Mann-Whitney U tests. *: *p* < 0.05.

To examine the molecular mechanism by which aerobic exercise suppressed cancer cachexia-induced muscle atrophy, we analyzed the expression of phosphorylated Akt (p-Akt), which mediates protein synthesis, by western blotting. The results showed that the expression of p-Akt is suppressed in the C26 bearing mice, and aerobic exercise increased the level of p-Akt expression in TA muscle (Figure 3A,B). Next, the downstream signaling pathways were analyzed by detecting the phosphorylated form of mTOR, p70S6 kinase, and 4EBP-1, which are involved in the protein synthesis signaling. The phosphorylated moiety of these proteins decreased under the cachectic condition and recovered by forced treadmill running, suggesting that aerobic exercise promoted de novo protein synthesis in the muscle tissues (Figure 3).

### 2.3. Aerobic Exercise Increases mRNA Expression of Adiponectin and Adiponectin-Related Genes in Skeletal Muscle

**Figure 4:**
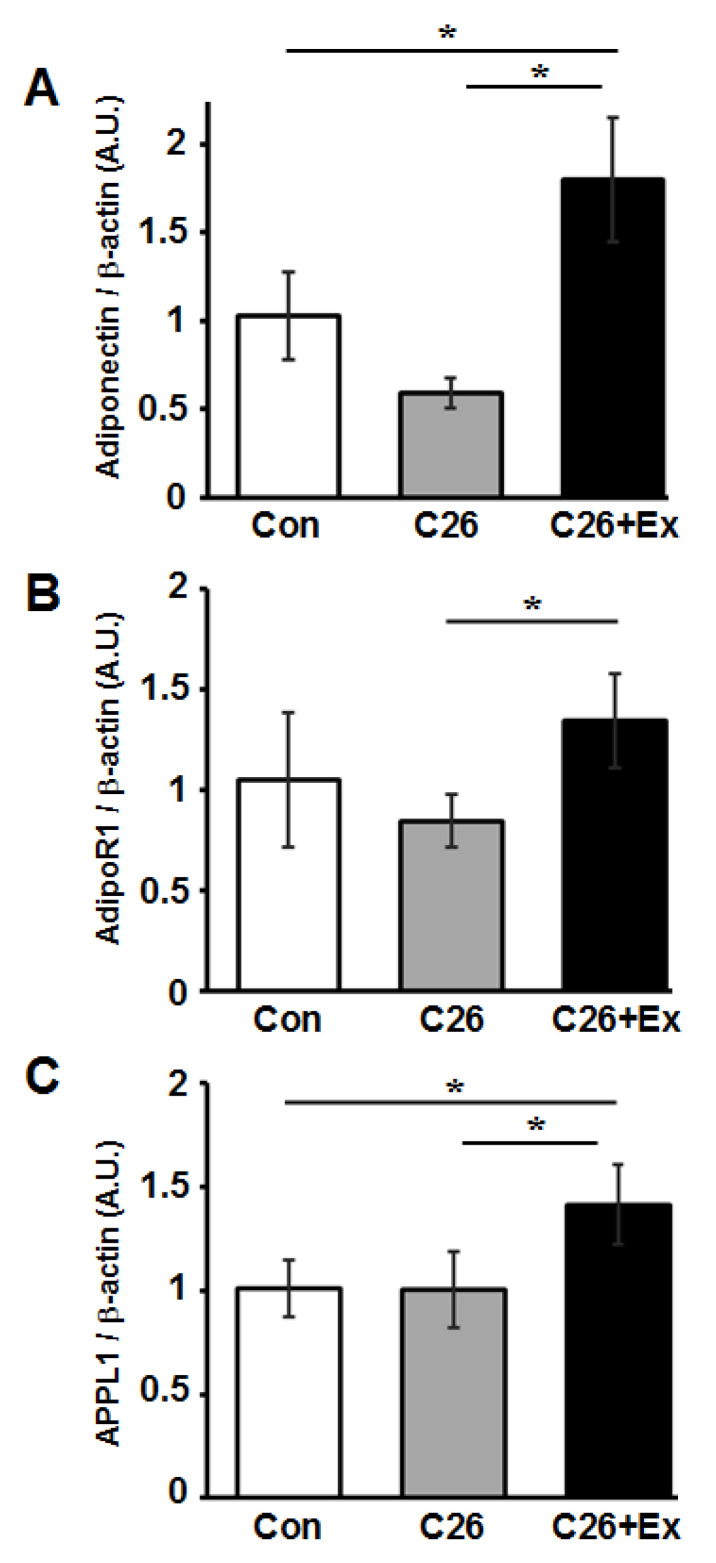
Increased expression of adiponectin and its related genes caused by forced aerobic exercise. Expression of (**A**) adiponectin, (**B**) AdipoR1, and (**C**) APPL1 in TA of Con, C26, and C26+Ex mice was examined by quantitative real-time PCR. The bar graphs indicate the mean relative expression ± SD. Statistical analysis was carried out with ANOVA followed by Tukey HSD tests. *: *p* < 0.05.

Wang et al. showed that adiponectin, an adipose-derived adipokine, induced elevated p-Akt levels in skeletal muscle tissues [37]. Therefore, we postulated that the recovery of p-Akt expression through aerobic exercise could be a downstream effect of adiponectin stimulation. To examine the mRNA expression of adiponectin-related genes, including adiponectin, AdioR1, and APPL1 during aerobic exercise, the mRNA level for these genes in TA muscles of control, C26, and C26+Ex group mice was examined by quantitative RT-PCR analysis. The expression of adiponectin, AdipoR1, and APPL1 was found to be significantly increased by aerobic exercise (Figure 4) (*p* < 0.05). 

### 2.4. Activation of Cellular Signaling by Recombinant Adiponectin in C2C12 Myotubes

To examine whether adiponectin can reduce muscle fiber atrophy, we used an in vitro cancer cachectic atrophy model and treated it with mouse recombinant adiponectin. The full-length mouse recombinant adiponectin that lacks its N-terminal signal sequence was expressed using a low-temperature inducible, bacterial expression system and purified with chelating metal chromatography (Figure S3).

**Figure 5:**
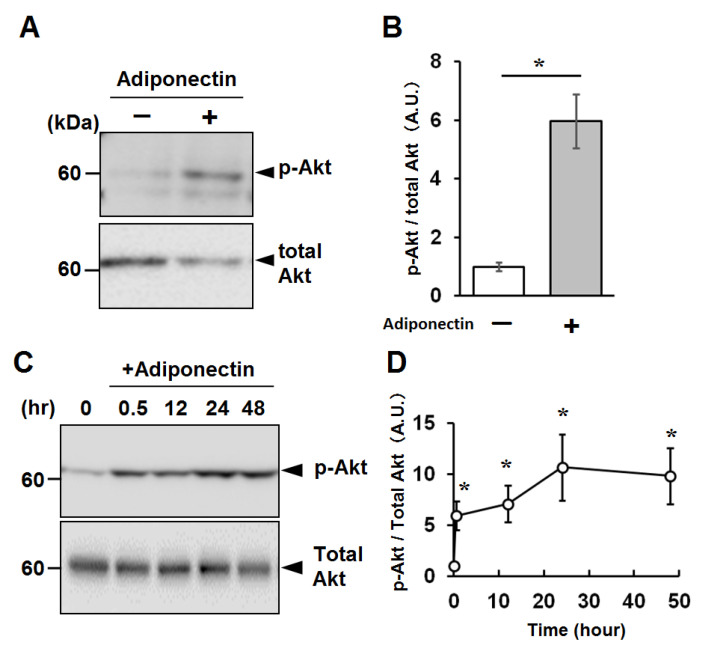
Recombinant adiponectin promotes Akt phosphorylation in C2C12 myotubes. (**A**) Pre-starved C2C12 myotubes were incubated in the absence (−) or presence (+) of 1 µg/mL recombinant adiponectin for 5 min, and cellular proteins were analyzed by western blotting using anti-phospho-Akt (p-Akt) and pan-Akt (Total) antibodies. A representative result from one of three independent experiments is shown. (**B**) Densitometric analysis of the western blot in (**A**). The mean ratio of phospho-Akt/total Akt ± SD is indicated. The Student’s *t*-test was used for statistical analysis. *: *p* < 0.05. (**C**) Time course of the Akt stimulation of C2C12 myotubes. C2C12 myotubes cultured in the presence of 1 µg/mL recombinant adiponectin for the indicated periods were subjected to western blotting as in (**A**). A representative result from one of three independent experiments is shown. (**D**) Densitometric analysis of p-Akt and total Akt signals in (**C**). The mean ratio of phospho-Akt/total Akt ± SD is indicated. Statistical analysis was performed by ANOVA, followed by Tukey HSD tests. *: *p* < 0.05, compared with adiponectin 0 hr (Adiponectin (−) in (**C**)) group.

To confirm the biological activity of the recombinant adiponectin, we examined the phosphorylation of Akt by western blotting. Treating C2C12 myotubes with 1 µg/mL recombinant adiponectin resulted in a significant elevation of phosphorylated Akt (Figure 5A,B). To further characterize the capacity of the recombinant adiponectin to stimulate myotubes, C2C12 myotubes were treated with adiponectin for various durations, and the adiponectin activity to stimulate Akt phosphorylation was observed for at least 48 h (Figure 5C,D).

### 2.5. Recombinant Adiponectin Ameliorates the Atrophy of Myotubes

**Figure 6:**
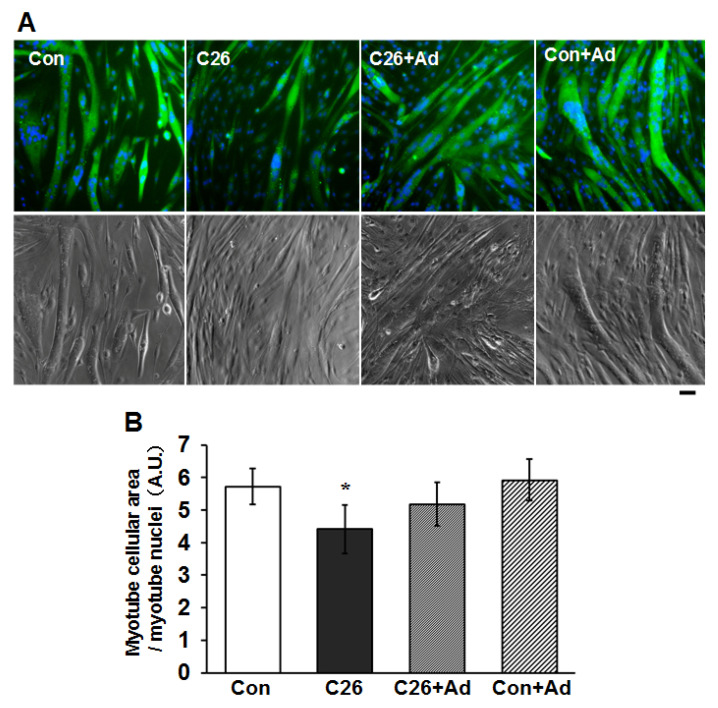
Adiponectin reverses the atrophy of myotubes caused by C26 conditioned medium. (**A**) Upper panels: C2C12 myotubes were treated with C26 derived conditioned medium (C26-CM) or 1 µg/mL recombinant adiponectin as indicated for 48 h, and the cellular area of myotubes was visualized by MYH4 immunostaining (green). Nuclei were stained by Hoechst33342 (blue). Phase-contrast images of the corresponding area are shown in the lower panels. Bars: 50 µm. (**B**) Relative multinucleated myotube areas per number of nuclei in the myotubes in (**A**) was analyzed. The bar graphs indicate the mean relative area ± SD. Statistical analysis was carried out using One-way ANOVA followed by Tukey HSD tests. *: *p* < 0.05, compared with control.

To examine whether the recombinant adiponectin might have a protective effect on cancer cachexia-induced muscle atrophy, we used an in vitro muscle atrophy model using C2C12 myotubes exposed to C26-conditioned medium (C26-CM). The addition of C26-CM to the C2C12 myotubes caused a decrease in the area of myotubes at 2 days compared to control (Figure 6), indicating that the atrophy of C2C12 myotubes was ameliorated by the addition of recombinant adiponectin.

### 2.6. Adiponectin Treatment Alters the Protein Degradation/Synthesis Balance in Myotubes

**Figure 7:**
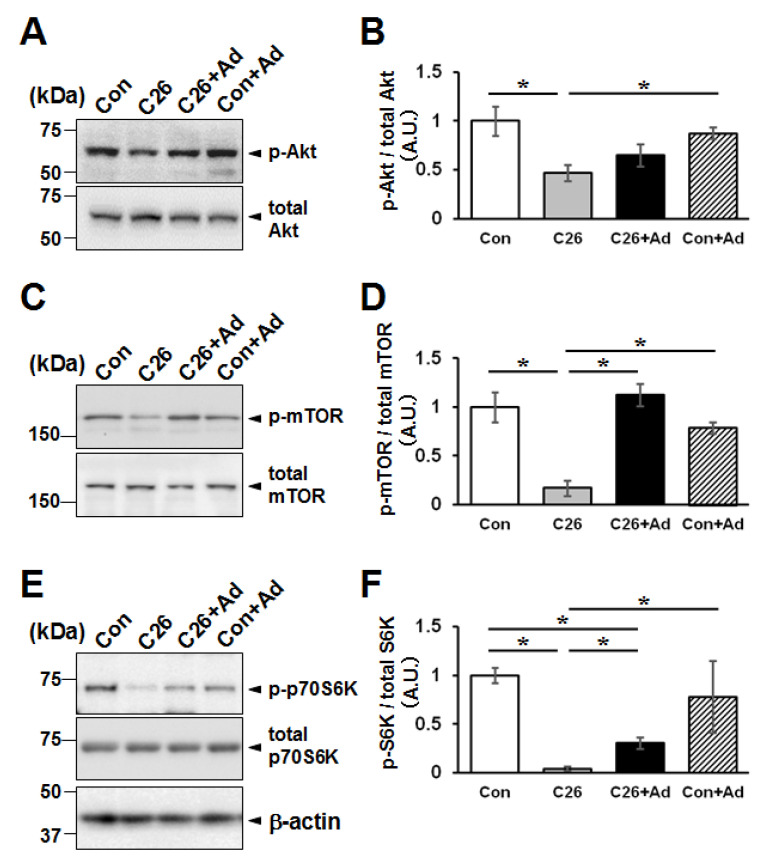
Effects of adiponectin treatment on the protein synthesis pathways in the in vitro cancer cachexia model. C2C12 myotubes were treated with C26 derived conditioned medium (C26-CM) or 1 µg/mL adiponectin, as indicated in Figure 6. Total proteins were analyzed by western blotting using antibodies for phospho-Akt and pan-Akt (**A**), phospho-mTOR and pan-mTOR (**C**), and phospho- and pan-p70S6 kinase (S6K) (**E**). A representative result from one of three independent experiments is shown. Densitometric analyses of western blotting shown in (**A**,**C**,**E**) are presented in (**B**,**D**,**F**) respectively. The bar graphs indicate the mean relative expression ± SD. Statistical analysis was performed by Kruskal-Wallis analysis followed by Mann-Whitney U tests. *: *p* < 0.05.

Using the C26-CM-dependent cellular atrophy model, we examined the effect of adiponectin treatment on phosphorylated mTOR (p-mTOR) expression, which is known to reflect the status of protein synthesis downstream of Akt phosphorylation [38]. The expression of p-mTOR and p-Akt decreased in C2C12 myotubes treated with C26-CM compared with control cells, and treatment with adiponectin increased p-Akt and p-mTOR levels in the C26-CM treated myotubes (Figure 7A–D).

Next, the phosphorylation of p70S6 kinase, a downstream signaling molecule involving the protein synthesis signals, was analyzed. As shown in Figure 7E, C26-CM treatment caused a decrease of p70S6 kinase phosphorylation level, and additional adiponectin treatment increased the phosphorylation level. Moreover, the net protein synthesis was examined by an in vivo puromycin labeling method, the SUnSET method [39]. The bulk puromycin signal decreased by C26-CM treatment, and adiponectin treatment suppressed the decrease and phosphorylation state of p70S6 kinase (Figure S4).

**Figure 8:**
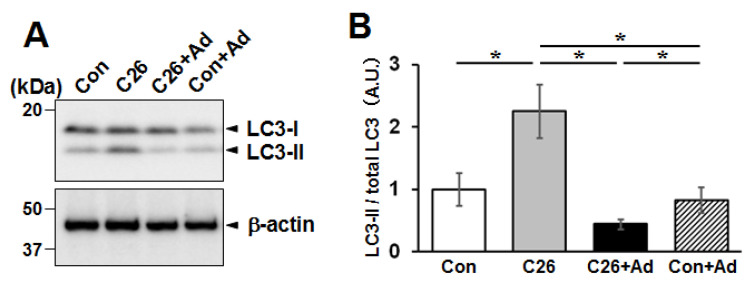
Effects of adiponectin treatment on LC3 expression. C2C12 myotubes were treated with C26-CM or adiponectin, and total proteins were analyzed by western blotting with anti-LC3 (**A**). Representative results from one of three independent experiments are shown. (**B**) Densitometric analyses of western blotting shown in (**A**) are presented in (**B**). The bar graphs indicate the mean LC3-II expression normalized by total LC3 (LC3-I+II) ± SD. Statistical analysis was performed by Kruskal-Wallis analysis followed by Mann-Whitney U tests. *: *p* < 0.05.

We next examined the expression of microtubule-associated protein 1A/1B-light chain 3 (LC3) by western blotting. In C26-CM-treated cells, there was a greater expression of LC3-II, the lipidated form of LC3, compared to that in the untreated control, suggesting the activation of autophagy [40]. Moreover, in both the control and C26-CM groups, treatment with adiponectin suppressed the expression of LC3-II (Figure 8A,B).

## 3. Discussion

### 3.1. Activation of Protein Synthesis Signal by Aerobic Exercise during Cancer Cachexia

In this study, we used the C26 bearing mouse cachexia model to investigate the effect of aerobic exercise on cachectic muscle atrophy. Unlike previous reports with a similar experimental system, total body weight did not change significantly after C26 injection, and no mice died during the 4-week study. In contrast, many previous studies with a similar experimental design showed a drastic loss of mice by cachexia during the 4-week experiments [20,21]. Notably, skeletal muscle wasting occurred as expected in our experimental conditions, suggesting that our C26 bearing mice were moderately cachectic. Therefore, we can focus on muscle atrophy more specifically without other indirect effects such as systemic inflammation [41].

Previous studies have demonstrated that aerobic exercise suppresses muscle atrophy in mice with cancer [16,20,21]. Here, we successfully reproduced those results and showed that aerobic exercise by treadmill running could suppress cancer cachexia-induced muscle atrophy. It has been reported that the atrophy of fast-twitch skeletal muscle occurs earlier than that of the slow-twitch skeletal muscle [42]. Therefore, the small muscle fibers observed in cancer cachectic skeletal muscle could be fast-type muscle fibers. Although the exercise-dependent protective effect was observed in the TA and GA, fast-twitch fiber dominant muscles, but not in the slow-twitch fiber dominant SOL, a precise experiment to distinguish the fiber types is needed to test the effect of exercise on specific slow and fast-twitch fibers.

The ubiquitin-proteasome system and autophagy are the primary mechanisms of cancer cachexia-induced muscle wasting [7,8,9,10,11]. In many cases, the elevation of the expression of muscle-specific E3 ligases, including MuRF1 and Atrogin-1, has been reported during cachexia [7,8,9,16,20,21]. In our study, however, we did not observe an increase in the expression of MuRF1 and Atrogin-1 in cachectic skeletal muscles (Figure S4). Moreover, we observed decreased expression of the phosphorylated form of Akt, mTOR, p70S6K, and 4EBP1, suggesting that de novo protein synthesis was affected under the cachectic condition.

In our current study, exercised cachectic mice did not show significant decrease of LC3-II, suggesting that aerobic exercise could not suppress activation of autophagy in skeletal muscle (Figure S5). Pigna et al. reported that aerobic exercise to C26 bearing mice counteracted cachectic muscle atrophy, maintaining the basal level of autophagy [20]. Moreover, they showed that aerobic exercise led to complete suppression of LC3-II, as opposed to the unchanged LC3-II levels observed in our studies. One significant difference between the data of the two groups is probably due to the difference in balance between the extent of cachexia and exercise intensity. Namely, an exercise program of forced treadmill running was applied to very moderate cachectic mice in our study. In that condition, exercise may provide stress response signals that could promote autophagy to some extent [43]. In contrast, Pigna et al. applied the voluntary wheel running to the mice with highly progressive cachexia made by C26 tumor graft [20]. Thus, it was possible that forced treadmill running benefited the mice in the early stages of cachexia, despite the risk of muscle injury. Therefore, the types and intensity of exercise should be carefully determined to effectively infer what clinical interventions can be prescribed to protect muscles from wasting in cancer cachexia.

### 3.2. Protective Effect of Adiponectin on Cancer Cachexia-Induced Muscle Atrophy

Insulin resistance is a known significant symptom of cancer cachexia [1]. Adiponectin has been reported to enhance insulin signaling through its specific receptor AdipoR1 and its downstream effectors, including APPL1 [22,23,25,26,27,28,29,30]. Therefore, we hypothesized that adiponectin might be involved in the protective effect of aerobic exercise against cachectic muscle atrophy. Indeed, aerobic exercise has been shown to significantly increase mRNA expression of adiponectin, AdipoR1, and APPL1 in the skeletal muscles of healthy mice, suggesting that adiponectin signaling molecules are up-regulated by aerobic exercise in vivo [44].

Moreover, in vitro analysis of the C2C12 atrophy model treated with adiponectin showed that the suppression of cellular atrophy was associated with the up-regulation of the Akt signaling pathway toward de novo protein synthesis through sequential activation of downstream mTOR and p70S6 kinase. It is well known that activation of mTOR inhibits autophagy [38]. In our results, indeed, down-regulation of LC3-II level was observed by adiponectin treatment, possibly due to activation of mTOR.

In contrast, adiponectin is also known to activate AMPK through AdipoR1 and AdipoR2 [28]. Besides the mTOR-dependent pathway, AMPK activation can induce autophagy [38]. It was also reported that AICAR, a specific activator for AMPK and rapamycin, an inhibitor of mTOR, could successfully suppress the cancer cachectic muscle atrophy [20]. At this moment, molecular regulatory mechanisms that coordinate these two pathways remain to be elucidated. Thus, further investigations into how these pathways protect against cachectic muscle wasting are needed.

### 3.3. Limitations and Conclusions

In the current study, we proposed that the protective effect of adiponectin on cancer cachectic muscle atrophy is through its alteration of the balance of protein synthesis and degradation. However, the study still has some significant limitations that hinder us from making any definitive conclusions. One problem is that the total experimental size was so small that the data could not be conclusive. Moreover, the severity of cachexia was so mild that direct comparison of our results with other previous reports was difficult. Our C26 bearing mice did not develop the major hallmarks for cachexia, such as decreased total body weight and induction of skeletal muscle atrogenes; however, skeletal muscle atrophy was clearly observed. Thus, our mouse model may be in early cachexia, the stage where clinical interventions would be effective.

In early cachectic stages, multifarious treatments, including aerobic exercise, have been recommended in clinical settings [45,46]; however, an effective exercise protocol has not been established. Therefore, uncovering the details of the mechanisms underlying the protective effect of aerobic exercise on the early stage of cancer cachexia-induced muscle atrophy is necessary for establishing effective exercise therapy. Our current study revealed that adiponectin could play a role in the protection mechanisms of aerobic exercise against muscle atrophy in cancer cachexia. Thus, this multifunctional cytokine is a potential target for rehabilitation and pharmaceutical treatments in patients with cancer cachexia.

## 4. Materials and Methods

### 4.1. Animals

All experiments and protocols were approved by the Nagoya University Animal Experiment Committee (permission number #31-019). Female wild type BALB/c mice, 10–11 weeks old, were purchased (CLEA Japan, Inc., Tokyo, Japan), allowed to acclimatize for 7 days, and then housed four mice per cage in an environmentally controlled room (22–24 controlled dark-light cycles of 12 h and allowed access to food and water ad libitum).

### 4.2. Cell Culture

C26 colon cancer cells and C2C12 myoblast cells were purchased from RIKEN Cell Bank (Tsukuba, Japan, #RCB2657) and ATCC (Manassas, VA, USA, #CRL1772), respectively. C26 cells were cultured in RPMI 1640 medium (Wako, Osaka, Japan) supplemented with 10% fetal bovine serum (FBS, COSMO Bio, Tokyo, Japan) and 1% penicillin-streptomycin (Sigma-Aldrich, St. Louis, MO, USA), while C2C12 cells were cultured in Dulbecco’s modified eagle medium (DMEM, Wako, Osaka, Japan) supplemented with 15% FBS and 1% penicillin-streptomycin in a 5% CO_2_ humidified atmosphere at 37 °C. C2C12 cells were differentiated into myotubes in DMEM containing 2% FBS for 5 days.

### 4.3. Injection of C26 into Mice

C26 cells were harvested using trypsin at 90–100% confluency, counted, and adjusted to 1 × 10^7^ cells/mL in phosphate-buffered saline (PBS, Sigma-Aldrich). Mice were anesthetized by intraperitoneal administration of pentobarbital (somnopentyl, 50 mg/kg body weight; Kyoritsu Seiyaku Corp., Tokyo, Japan), and 100 µL of the C26 suspension (1 × 10^6^ cells) was subcutaneously injected into the back of the mice. In the control group, 100 µL of PBS was injected.

### 4.4. Animal Experimental Design

Mice were randomly assigned to control (*n* = 4), cancer cachexia (C26, *n* = 4), and cancer cachexia with aerobic exercise (C26+Ex, *n* = 4) groups. C26 cells were subcutaneously injected into the back of the mice in the C26 and C26+Ex groups. Mice in the C26+Ex group were forced to run on a treadmill for 4 weeks after C26 injection, as described in 4.5. Four weeks after the injection, mice in all groups were sacrificed, and their skeletal muscles were harvested for gene expression analysis.

### 4.5. Aerobic Exercise

The mice were habituated to the treadmill for 4 to 5 days before the test. Habituation was performed by first placing the mice on a stationary band for a few minutes. The speed was then gradually increased to ensure that the mice were familiarized with running on the treadmill. From the day after the injection of C26 cells, mice in the C26+Ex group were subjected to treadmill running for 30 min/day, 5 days/week, for 4 weeks. The running speed was 12 m/min, and the experiment was conducted in the dark cycle. The treadmill used for the exercise was made in the in-house facility and was equipped with a speed controller (US590-01CT, Oriental motor corporation, Tokyo, Japan). Detailed information about the treadmill can be provided upon request.

### 4.6. Collection of Tissues

Mice were sacrificed 4 weeks after the day of injection. Tumors, tibial anterior (TA) muscle, gastrocnemius (GA), and soleus (SOL) muscles were collected, and their weights were measured. The left hind limb muscles were collected for histological analysis, while the right hind limb muscles were harvested for mRNA and protein sampling. The isolated hind limb muscles were cut in half and stored at −80 °C until analysis.

### 4.7. Analysis of Muscle Cross-Sectional Area

Muscle tissue isolated from the left hind limb was stuck to a corkboard piece using Tragacanth gum (Wako). Then, the skeletal muscle was rapidly frozen in isopentane (Wako) pre-chilled in liquid nitrogen and stored at −80 °C until sectioning. Seven µm-thick cross-sections were made from muscle samples with a cryostat (Leica, Wetzlar, Germany) at −20 °C, fixed with 4% paraformaldehyde (Sigma-Aldrich) in PBS, and then stained with Alexa555-conjugated phalloidin (Thermo Fisher Scientific, Waltham, MA, USA) and Hoechst 33342 (Sigma-Aldrich). After staining, the cross-sections were washed with PBS and mounted using Entellan new (MERCK, Darmstadt, Germany). Stained muscle sections were observed on a fluorescence microscope (BZ-9000: KEYENCE, Osaka, Japan), and captured fluorescent images of the cross-sections were analyzed using the ImageJ software (Fiji package; NIH, Bethesda, MD, USA).

### 4.8. Western Blotting

TA muscle cut in half was homogenized in 200 µL of lysis buffer [5 mM EDTA (Sigma-Aldrich), 1× Protease Inhibitor Cocktail (PIC, Roche Diagnostics, Mannheim, Germany), 1 mM β-glycerophosphate (Kanto Chemical Co., Inc., Tokyo, Japan), and 1 mM sodium orthovanadate (Wako)]. Then, the suspension was sonicated on ice after adding 10 µL of 10% SDS (Wako). The sonicated sample was centrifuged at 17,400× *g* for 10 min, and the supernatant was recovered. The protein abundance of each sample was quantified using the bicinchoninic acid (BCA) assay adjusted to 1 mg/mL. C2C12 cells were lysed in the sample buffer. The collected sample was re-sonicated on ice, boiled at 90 °C for 5 min, and subjected to SDS-PAGE. Then, the separated proteins were transferred to a polyvinylidene fluoride (PVDF) membrane (MERCK).

Subsequently, 3% bovine serum albumin (BSA, Wako) or skimmed milk was used to block nonspecific reactions for 1 h at 25 °C. Then, membranes were incubated at 4 °C overnight with primary antibody diluted in PBS containing 0.1% (*w*/*v*) Tween 20 (Wako) (T-PBS). The following primary antibodies and dilutions were used: p-Akt (pSer473; 1:1000; Cell signaling Technology, Danvers, MA, USA, #4060), Akt (1:1000; Cell signaling technology, #9272), p-mTOR (pSer2448; 1:1000; Cell signaling technology, #5536), mTOR (1:1000; Cell signaling technology, #2972), p-p70S6K (pThr389; 1:1000; Cell signaling technology #9234), p70S6K (1:1000; Cell signaling technology #2708), p-4EBP-1 (pSer65; 1:1000; Cell Signaling Technology, #9451), 4EBP (1:1000; Cell signaling technology #9452), LC3 (1:250; rabbit polyclonal antibody raised against rat LC3B, kindly provided by Prof. Ueno, Juntendo University), β-actin (1:5000; Proteintech, Tokyo, Japan, #60008-1-Ig). Then, the membranes were washed with T-PBS and incubated for 1 h at 25 °C with HRP-conjugated anti-mouse or rabbit IgG (1:10000; Cytiva, Tokyo, Japan) secondary antibodies diluted in T-PBS.

After extensive washing with T-PBS, immunoreactive signals were detected by chemiluminescence reagent (Takara Bio Inc., Shiga, Japan) and EZ-capture (AE-9160PH: ATTO Corporation, Tokyo, Japan), and the signals were quantified using the ImageJ software. β-actin was used as an internal standard.

### 4.9. Real-Time RT-PCR

For RNA sample preparation, each TA muscle cut in half was homogenized in 100 µL of TRI Reagent (Cosmo Bio) and incubated at 4 °C overnight; total RNA was purified from the homogenate following manufacturer’s instructions. Accordingly, 1 µg of total RNA was converted to cDNA using the 5× All-In-One MasterMix (Applied Biological Materials Inc., Richmond, BC, Canada). Real-time RT-PCR was performed using the Step One Plus^®^ Real-time PCR system (Thermo Fisher Scientific) and SYBR^®^ Green Realtime PCR Master Mix (Toyobo, Osaka, Japan).

The following primers were used: MuRF1 (fwd: 5′-ACGGAAACGACCTCCAGACATG-3′, rev: TACCAAGCCTGTGGTCATCCTG-3′), Atrogin-1 (fwd: 5′-CTTCTCGACTGCCATCCTGGAT-3′, rev: 5′-TCTTTTGGGCGATGCCACTCAG-3′), Myostatin (fwd: 5′-AGTGGATCTAAATGAGGGCAGT-3′, rev: 5′-GTTTCCAGGCGCAGCTTAC-3′), Adiponectin (fwd: 5′-GGAGTGTTCGTGGGCTTAGG-3′, rev: 5′-GCAGCTCCGGTGATATAGAGG-3′), AdipoR1 (fwd: 5′-ACGTTGGAGAGTCATCCCGTAT-3′, rev: 5′-CTCTGTGTGGATGCGGAAGAT-3′), APPL1 (fwd: 5′-AGCCATGACCCTTTATATCTGC-3′, rev: 5′-AGGTATCCAGCCTTTCGG-3′), and β-actin (fwd: 5′-GGAGCAGATTAGTAAGCGGCTTG-3′, rev: 5′-GTTACTGCCACAGGAACTAGAGG-3′).

### 4.10. Expression and Purification of Recombinant Adiponectin

The mouse adiponectin open reading frame deprived of the first 60 bp, which corresponds to the signal sequence, was amplified by PCR and cloned into the pCold-I vector *Eco*RI and *Xho*I sites (Takara Bio Inc.). The resultant plasmid, pColdI-His-mAdiponectin, was transformed into the DH5α bacterial strain (Toyobo).

For induction of the recombinant protein, logarithmic phase bacterial culture was shifted to 15 °C, and IPTG (Sigma-Aldrich) was added to a final concentration of 1 mM, followed by further culturing for 12 h. Bacterial cells were harvested by centrifugation at 8900× *g* for 15 min and sonicated in PBS containing 1× proteinase inhibitor cocktail (Sigma-Aldrich) at 4 °C. The cell homogenate was centrifuged at 17,400× *g* for 15 min to remove debris, and Talon beads (Takara Bio Inc.) were added to the soluble fraction. After 12 h of mixing, Talon beads were washed extensively with PBS, and the bound His-tagged adiponectin was eluted with 20 mM imidazole (Wako) in PBS.

Purified protein samples were subjected to endotoxin removal using the ToxinEraser™ Endotoxin Removal Kit (GenScript Biotech, Piscataway, NJ, USA) following the manufacturer’s protocol.

### 4.11. In Vitro Cancer Cachexia-Induced Muscle Atrophy Model

C26-conditioned medium (C26-CM) was prepared by culturing confluent C26 cells in serum-free DMEM for 48 h. The C26-CM medium was centrifuged (5000× *g*, 4 °C, 5 min) to remove debris. Then, C26-CM was diluted with DMEM supplemented with 2% FBS and added simultaneously with recombinant adiponectin to C2C12 myotubes to induce muscle atrophy-related reactions. At 48 h after the addition of C26-CM and adiponectin, C2C12 was lysed using sample buffer, and protein expression was analyzed. For morphological analysis of the myotubes, cells were fixed in 4% paraformaldehyde in PBS for 10 min, followed by treatment with PBS containing 0.1% TritonX-100 (Sigma-Aldrich). The cells were then stained with anti-MYH4 (1:2000, Thermo Fisher Scientific, MF20) and Hoechst 33342 (Sigma-Aldrich). Cell images were taken using BZ-9000 microscopy (KEYENCE) and were analyzed using ImageJ software (NIH, Bethesda, MD, USA).

A nonisotopic-labeled protein synthesis assay (SUnSET method) was performed as described previously [39]. In brief, puromycin (0.04 μM) was added to the culture medium 60 min before harvesting the cells. Protein was extracted and analyzed as stated in Section 4.8. Total protein was assessed with Coomassie blue staining, and puromycin-incorporated newly synthesized protein was detected by western blotting using a puromycin antibody (1:2000, CosmoBio).

### 4.12. Statistical Analysis

All data are presented as mean ± standard deviation and were compiled from at least two independent experiments performed in triplicate. *p*-values were calculated by nonparametric tests: Kruskal-Wallis followed by Mann-Whitney U tests, or One-way ANOVA followed by Tukey HSD tests, as indicated. Statistical analysis was performed using SPSS software (version 2.7; IBM Corp., Armonk, NY, USA). Values were considered statistically significant at *p* < 0.05.

## Supplementary Materials

The following are available online at https://www.mdpi.com/1422-0067/22/6/3110/s1, Figure S1. Histogram of the CSA of muscle fibers, Figure S2. Effect of aerobic exercise on tumor size, Figure S3. Bacterially expressed histidine tagged-mouse adiponectin was purified and subjected to SDS-PAGE and CBB staining, Figure S4. C2C12 myotubes treated with C26 with or without recombinant adiponectin for 48 hours as described as figure 7 in the text, Figure S5. Effect of aerobic exercise on expression of protein degradation related genes.
