# Supplementary material for: Aerobic Exercise Ameliorates Cancer Cachexia-Induced Muscle Wasting through Adiponectin Signaling
Source: Int J Mol Sci. 2021 Mar 18;22(6):3110. doi: 10.3390/ijms22063110 (PMC8002946; doi:10.3390/ijms22063110)
Supplement: Supplementary file 1 [file ijms-22-03110-s001.pdf]

Supplemental Figures Morinaga et al. ijms-1035567

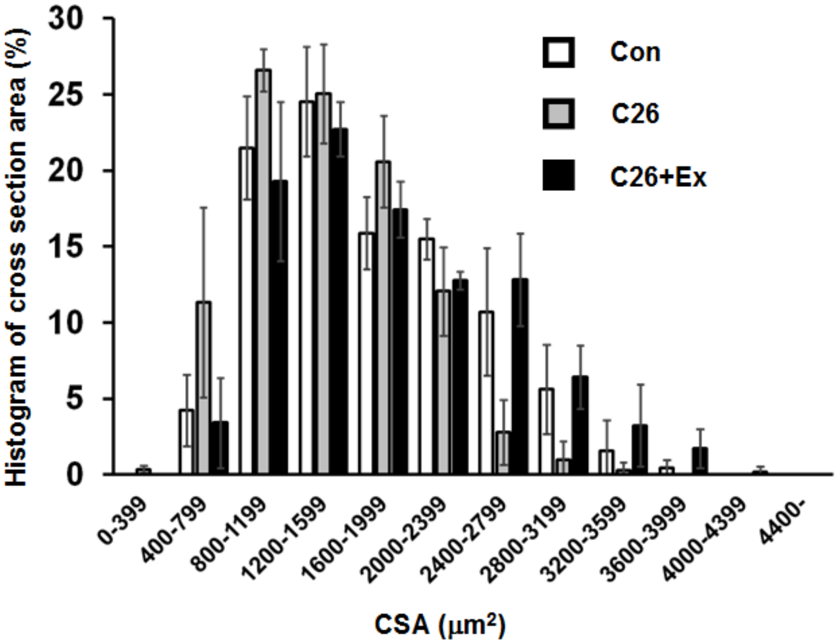

Supplemental Figure S1
Histogram of the CSA of muscle fibers. Cross sectional area of each muscle fiber from the TA of control (Con), C26-bearing mice (C26), and aerobic exercise-loaded C26-bearing mice (C26+Ex) is shown.

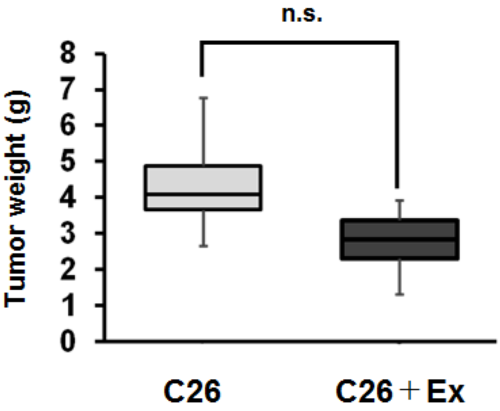

Supplemental Figure S2
Effect of aerobic exercise on tumor size. Weight of tumors from C26 and C26+Ex groups are indicated. n.s.: not significant

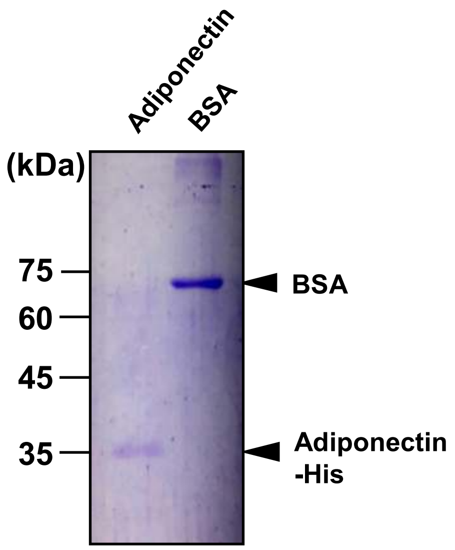

Supplemental Figure S3
Bacterially expressed histidine tagged-mouse adiponectin was purified and subjected to SDS-PAGE and CBB staining. One µg of bovine serum albumin (BSA) is indicated.

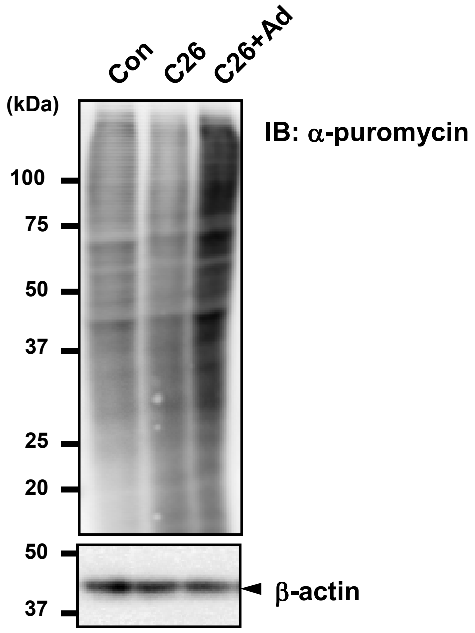

Supplemental Figure S4
C2C12 myotubes treated with C26 with or without recombinant adiponectin for 48 hours as described as figure 7 in the text. Cell were treated with 40 nM puromycin 60 min prior to cell lysis. Total lysate was subjected to western blotting using anti-puromycin antibody. β-actin is visualized as loading control.

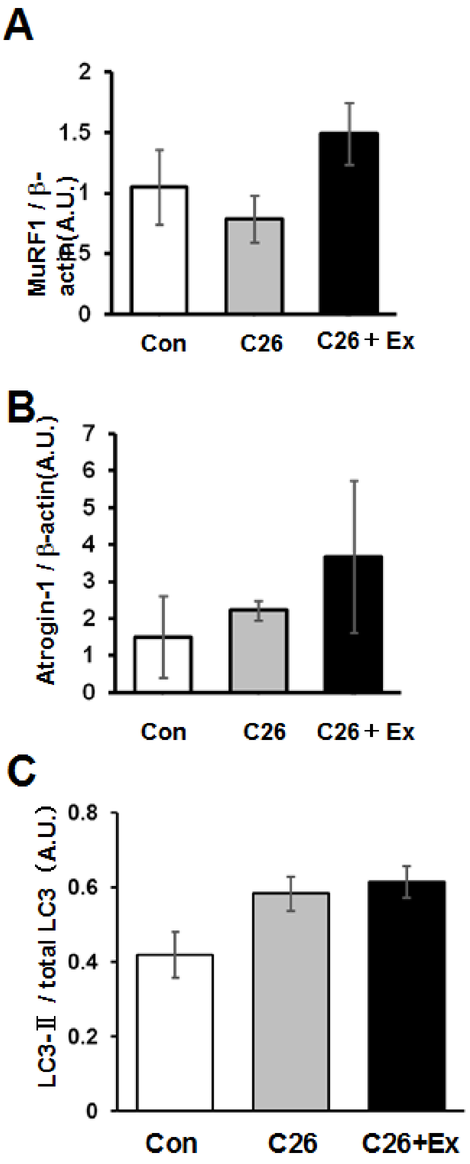

Supplemental Figure S5

Effect of aerobic exercise on expression of protein degradation related genes. Expression of MuRF1 (A) and Atrogin-1 (B) in the TA muscle of control (Con), cancer bearing (C26), C26 treated with aerobic exercise (C26+Ex) mice was examined by quantitative PCR. (C) Expression of LC3 II in the muscle tissues as in (A) was examined by immunoblotting. Relative expression level of LC3-II normalized by total LC3 (LC3I+II) is indicated.
